# Tissue engineering, stem cells, cloning, and parthenogenesis: new paradigms for therapy

**DOI:** 10.1186/1743-1050-1-3

**Published:** 2004-12-08

**Authors:** Jason Hipp, Anthony Atala

**Affiliations:** 1 Wake Forest Institute for Regenerative Medicine Wake Forest University School of Medicine Winston Salem, North Carolina USA; 2 Wake Forest University School of Medicine Medical Center Blvd. Winston Salem, North Carolina 27157 USA

## Abstract

Patients suffering from diseased and injured organs may be treated with transplanted organs. However, there is a severe shortage of donor organs which is worsening yearly due to the aging population. Scientists in the field of tissue engineering apply the principles of cell transplantation, materials science, and bioengineering to construct biological substitutes that will restore and maintain normal function in diseased and injured tissues. Both therapeutic cloning (nucleus from a donor cell is transferred into an enucleated oocyte), and parthenogenesis (oocyte is activated and stimulated to divide), permit extraction of pluripotent embryonic stem cells, and offer a potentially limitless source of cells for tissue engineering applications. The stem cell field is also advancing rapidly, opening new options for therapy. The present article reviews recent progress in tissue engineering and describes applications of these new technologies that may offer novel therapies for patients with end-stage organ failure.

## Introduction

The goal of tissue engineering is to repair organ pathologies such as those acquired congenitally or by cancer, trauma, infection, or inflammation. It is based upon the foundations of cell transplantation and materials science. Tissue can be engineered 1) *in vivo*- by stimulating the body's own regeneration response with the appropriate biomaterial, or 2) *ex vivo*- cells can be expanded in culture, attached to a scaffold and then reimplanted into the host. Cells may be heterologous (different species), allogeneic (same species, different individual), or autologous (same individual). Autologous cells are preferred because they will not evoke an immunologic response and thus the deleterious side effects of immunosuppressive agents can be avoided.

The ideal autologous cells can often be found within the organ itself. These cells (committed precursors) may be isolated, expanded and transplanted back into the same patient, thus representing an autologous transplantation resource. Previously, urothelial cells could be grown in the laboratory setting with only limited expansion. Several protocols were developed over the last 20 years which identified the undifferentiated cells and kept them undifferentiated during their growth phase [1-4]. Using such cell culture methods it is now possible to expand a urothelial strain from a single specimen which initially covered a surface area of 1 cm^2 ^to one that covers a surface area of >4000 m^2 ^(an area equivalent to one football field) within 8–14 weeks. These studies indicate the possibility of collecting autologous bladder cells from human patients, expanding them in culture, and returning them to the human donor in sufficient quantities for reconstructive purposes [1,3-11]. Major advances have been achieved within the past decade regarding possible expansion of several primary human cell types with specific techniques that employ autologous cells for clinical application.

While autologous cells are recognized as the ideal transplantation resource, many patients with end-stage organ disease are unable to yield sufficient cells for expansion and transplantation. Furthermore, some primary autologous human cells cannot be expanded from particular organs (*i.e*. pancreas, liver). Stem cells are envisioned as being an alternate source of cells from which the desired tissue can be derived. Human embryonic stem cells (HESC) can be derived from discarded non transferred embryos and have the advantage of being pluripotential (the ability to differentiate into all tissues of the embryo) and able to self-renew indefinitely. However, their clinical application is limited because they represent an allogeneic resource and thus their use would require high dose immunosuppressant therapy.

New stem cell technologies such as somatic cell nuclear transfer (therapeutic cloning) and parthenogenesis offer an exciting alternative to create an inexhaustible supply of ESC that can differentiate into all cell types of the embryo, while not being rejected by the patient's immune system. Although many tissues have been created with ESC, they are not used clinically because of an inability to control differentiation. Hence, their ability to form multiple tissue types also becomes their limitation. New genomics and bioinformatics technologies have and will continue to offer new insights into the understanding of ESC growth and differentiation and their application to engineering tissues. In the near future, these new technologies will allow for the generation of an unlimited supply of any cell type in the body.

### Stem cells

The political and ethical controversy surrounding stem cells began in 1998 with the creation of HESC derived from discarded, non-transferred human embryos[12]. The HESC were isolated from the inner cell mass of a blastocyst (5 days post-fertilization embryo) using an immunosurgical technique whereby the blastocyst was incubated with antibodies specific to trophectoderm. Complement proteins then resulted in lysis of the trophectoderm so that the only surviving cells were the inner cell mass [13]. Given that some cells can not be expanded *ex vivo*, ESC can potentially be the ideal resource for tissue engineering because of two fundamental properties, 1) the ability to self-renew indefinitely, and 2) the ability to differentiate into all three germ layers.

With the current restrictions surrounding HESC work, many proponents of stem cell research have sought to modify the ban to incorporate the thousands of non-transferred frozen embryos resulting from IVF to be used for the creation of more HESC lines. A SART-RAND study identified approximately 400,000 frozen embryos in storage since the late 1970s [14]. However, only 2.8% of these have been designated for research. Of the 11,000 embryos designated for research, only 65% of these (*n *= 7,334) are expected to survive the freeze/thaw process. From this, 25% are expected to develop to blastocyst stage (*n *= 1, 834). If one assumes a 15% efficiency rate for establishment of a HESC line from blastocysts (as suggested by previous studies [12,15]), it may be estimated that approximately 275 HESC could be created from excess frozen embryos. However, the real number of HESC line generated would actually be much lower since not all frozen embryos allocated for research would be used to create HESC lines. Furthermore, even if the maximum possible number of HESC lines could be derived from human frozen embryos, the clinical application of such cells would be limited by the potential rejection from another individual's immune system. New stem cell technologies (such as somatic cell nuclear transfer and parthenogenesis) promise to overcome this limitation.

### Somatic cell nuclear transfer (therapeutic cloning)

Somatic cell nuclear transfer (SCNT) entails the removal of an oocyte nucleus followed by its replacement with a nucleus derived from a somatic cell obtained from that patient. Activation with chemicals or electric shock stimulates cell division up to the blastocyst stage at which time the inner cell mass is isolated and cultured, resulting in ESC. This approach is distinct from reproductive cloning because the blasotcyst is not transplanted back to the uterus. Hence, development does not proceed beyond the 100 cell stage. This process also differs from fertilization since no sperm is used in this process. The resulting ESC are perfectly matched to the patients immune system and no immunosuppressants would therefore be required to prevent rejection.

While interest in the field of nuclear cloning remains high since the birth of Dolly (1997), the first successful nuclear transfer was actually reported over fifty years ago by Briggs and King [16]. Cloned frogs, which were the first vertebrates derived from nuclear transfer, were subsequently reported by Gurdon in 1962 [17] although the nuclei were derived from non-adult sources. Indeed, in just the past six years alone important advances in nuclear cloning technology have been reported – a pace of discovery that betokens the relative immaturity of this research arena. In fact Dolly was not the first cloned mammal to be produced from adult cells. Live lambs were produced in 1996 using nuclear transfer and differentiated epithelial cells, although these were derived from embryonic discs [18]. To be sure, the significance of the Dolly report was that this described the first mammal to be derived from an *adult *somatic cell using nuclear transfer [19]. Subsequently, animals from several species have been grown using nuclear transfer technology, including cattle [20], goats [21,22], mice [23], and pigs [24-27].

**Figure 1:**
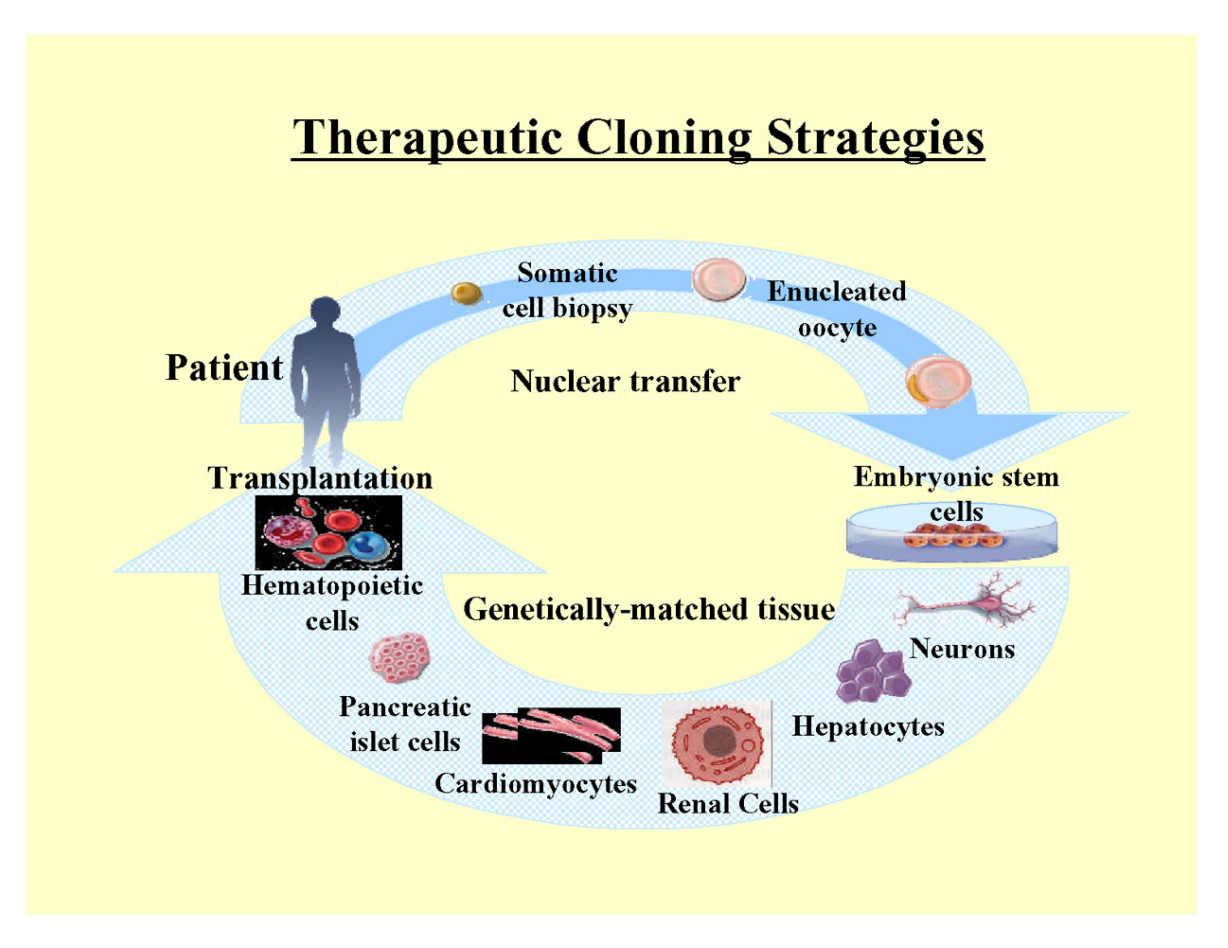
Strategy for therapeutic cloning and tissue engineering

A better understanding of the differences between reproductive cloning and therapeutic cloning may help alleviate some of the controversy surrounding these technologies [28,29]. Banned in most countries for human applications, reproductive cloning is used to generate an embryo that has the identical genetic material as its cell source. Such an embryo could then be implanted into the uterus of a female to give rise to a liveborn infant that is a clone of the donor. In contrast, therapeutic cloning is used to generate only ESC lines whose genetic material is identical to that of its source. These autologous stem cells have the potential to become almost any type of cell in the adult body, and thus would be useful in tissue and organ replacement applications [30]. Therefore, therapeutic cloning (SCNT) may provide an alternative source of transplantable cells. Figure 1 shows the strategy of combining therapeutic cloning with tissue engineering to develop tissues and organs. It has been estimated that approximately 3,000 people die every day in USA of diseases that could have been treated with stem cells-derived tissues [31]. With current allogeneic tissue transplantation protocols, rejection is a frequent complication because of immunologic incompatibility and thus immunosuppressive drugs are generally required to manage host-versus-graft disease [30]. The use of transplantable tissue and organs derived from therapeutic cloning could obviate unwanted immune responses typically associated with transplantation of non-autologous tissues [32].

While promising, somatic cell nuclear transfer technology has certain limitations requiring further improvement before it can be applied widely in clinical practice. Currently, the efficiency of the overall cloning process is quite low as the majority of embryos derived from animal cloning do not survive after implantation [33-35]. In practical terms, multiple nuclear transfers must be performed in order to produce one live offspring for animal cloning applications. The potential for cloned embryos to grow into live offspring ranges between <1 and 18% for sheep, pigs, and mice [36]. However, greater success (~ 80%) has been reported in cattle [37], a result which may in part be due to availability of advanced laboratory technologies specifically developed for this species for agricultural/breeding purposes. To improve cloning efficiencies, further improvements are required in the multiple complex steps of nuclear transfer such as enucleation and reconstruction, oocyte activation, and synchronization of cell cycle between donor cells and recipient oocytes [38].

It must be noted that abnormalities have been found in liveborn clones including macrosomia with an enlarged placenta ("large-offspring syndrome") [39], respiratory distress, defects of the kidney, liver, heart, and brain [40], obesity [41], and premature death [42]. These may be related to epigenetics of cloned cells which involve reversible modifications of DNA, while the original DNA (genetic) sequences remain intact. Faulty epigenetic modulation in clones may result from altered DNA methylation and/or histone modifications causing the overall chromatin structure of somatic nuclei not to be reprogrammed to an embryonic pattern of expression [30]. Reactivation of key embryonic genes at the blastocyst stage usually does not occur in embryos cloned from somatic cells, while embryos cloned from embryos consistently express early embryonic genes[43,44]. Proper epigenetic reprogramming to an embryonic state may help to improve the cloning efficiency and reduce the incidence of abnormal cloned cells.

### Novel applications of somatic cell nuclear transfer (therapeutic cloning)

We applied principles of both tissue engineering and therapeutic cloning in an effort to produce genetically identical renal tissue in an animal model (*Bos taurus*) [45]. Bovine skin fibroblasts from adult Holstein steers were obtained by ear notch and single donor cells were isolated and microinjected into the perivitelline space of donor enucleated oocytes (nuclear transfer). The resulting blastocysts were transferred to the uterus of progestin-synchronized recipients permit further *in vivo *growth. After 12 weeks cloned renal cells were harvested, expanded *in vitro*, then seeded onto biodegradable scaffolds. The constructs (consisting of cells + scaffolds) were then implanted into the subcutaneous space of the same steer from which the cells were cloned to allow for tissue growth.

The kidney is a complex organ with multiple cell types and a complex functional anatomy rendering it one of the most difficult organs to reconstruct [46,47]. Previous efforts in tissue engineering of the kidney have been directed toward development of extracorporeal renal support systems made of biological and synthetic components [48-54]. Although *ex vivo *renal replacement devices are known to be life-sustaining, there are obvious benefits for patients with end-stage kidney disease if such devices could be implanted long-term without the need for an extracorporeal perfusion circuit or immunosuppressive drugs.

**Figure 2:**
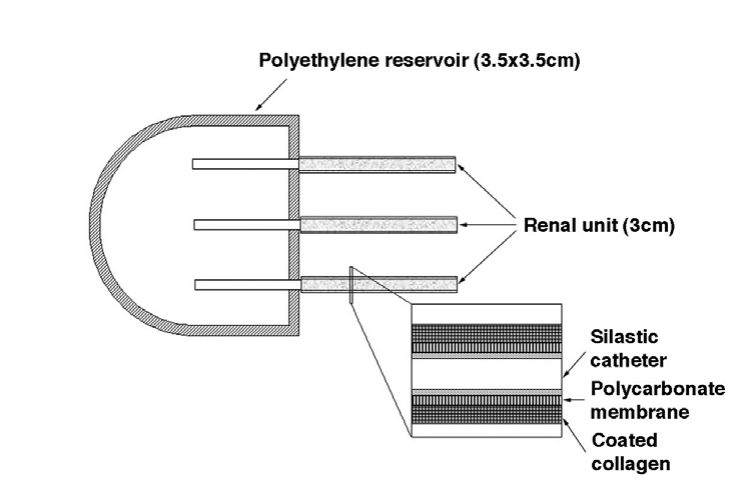
Combining therapeutic cloning and tissue engineering to produce kidney tissue, an illustration of the tissue-engineered renal unit.

**Figure 3:**
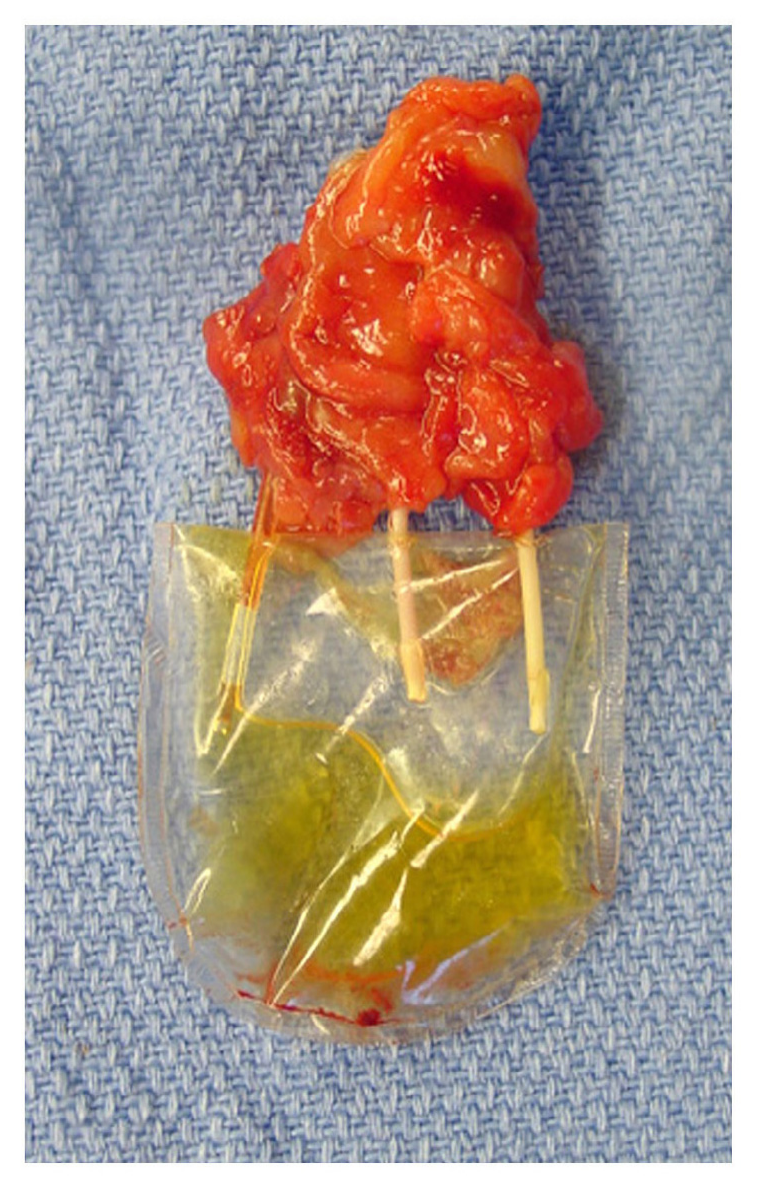
Renal unit seeded with cloned cells, three months after implantation, showing the accumulation of urinelike fluid.

Cloned renal cells were seeded on scaffolds consisting of three collagen-coated cylindrical polycarbonate membranes (figure 2). The ends of the three membranes of each scaffold were connected to catheters terminating in a collecting reservoir. This created a renal neo-organ with a mechanism for collecting the excreted urinary fluid (figure 3). Scaffolds with the collecting devices were transplanted subcutaneously into the same steer from which the genetic material originated and retrieved 12 weeks after implantation.

**Figure 4:**
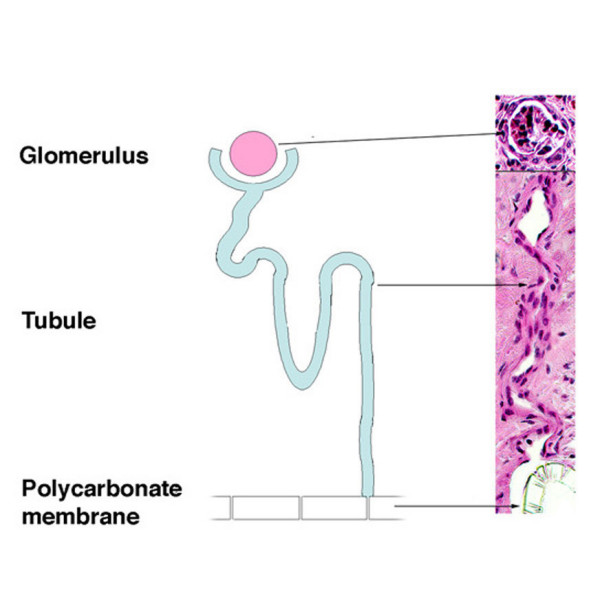
Clear unidirectional continuity between the mature glomeruli, their tubules, and the polycarbonate membrane.

Chemical analysis of the urine-like fluid (for urea nitrogen/creatinine levels, electrolyte levels, specific gravity, and glucose concentration) revealed that the implanted renal cells possessed filtration, reabsorption, and secretory capabilities. Histological examination of the retrieved implants revealed extensive vascularization and self-organization of the cells into glomeruli- and tubule-like structures. A clear continuity between glomeruli, tubules, and the polycarbonate membrane was noted that allowed the passage of urine into the collecting reservoir (figure 4). Immunohistochemical analysis with kidney-specific antibodies revealed the presence of renal proteins, and RT-PCR analysis confirmed the transcription of renal specific RNA in the cloned specimens. Western blot analysis confirmed the presence of elevated renal-specific protein levels.

As previous studies have confirmed bovine clones harbor mitochondrial DNA (mtDNA) of strictly oocyte origin [55-57], the donor egg's mtDNA was thought to be a potential source of immunologic incompatibility. Differences in mtDNA-encoded proteins expressed by cloned cells could stimulate a T-cell response specific for mt-DNA-encoded minor histocompatibility antigens when cloned cells are implanted back into the original nuclear donor [58]. We used nucleotide sequencing of the mtDNA genomes of the clone and fibroblast nuclear donor to identify potential antigens in the muscle constructs. Only two amino acid substitutions were noted to distinguish cells from the clone and the nuclear donor. Since peptide-binding motifs for bovine MHC class I molecules remain poorly understood, there is no reliable method to predict the impact of these amino acid substitutions on bovine histocompatibility.

**Figure 5:**
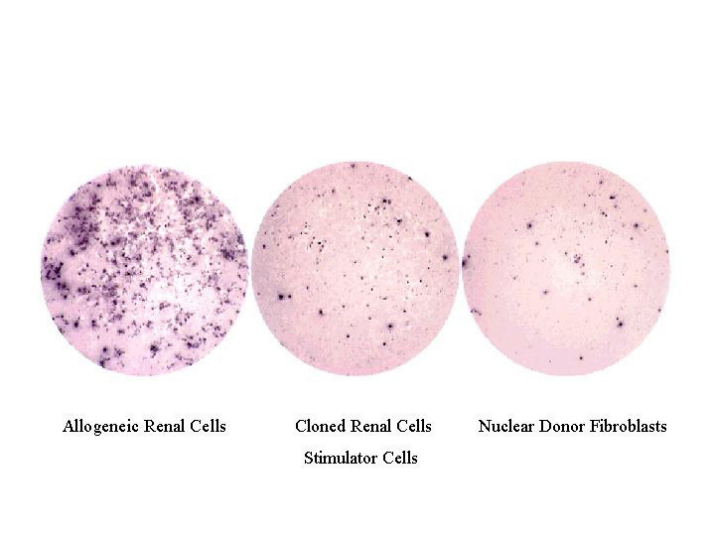
Elispot analyses of the frequencies of T-cells that secrete IFN-gamma after primary and secondary stimulation with allogeneic renal cells, cloned renal cells, or nuclear donor fibroblasts.

Oocyte-derived mtDNA was also considered to be a potential source of immunologic incompatibility in cloned renal cells. Maternally transmitted minor histocompatibility antigens in mice have been shown to stimulate both skin allograft rejection *in vivo *and cytotoxic T lymphocytes expansion *in vitro *[58] that could prevent the use of these cloned constructs in patients with chronic rejection of major histocompatibility-matched human renal transplants [59,60]. We tested for a possible T-cell response to the cloned renal devices using delayed-type hypersensitivity testing *in vivo *and Elispot analysis of interferon-gamma secreting T-cells *in vitro*. Both analyses revealed that the cloned renal cells showed no evidence of T-cell response, suggesting that rejection will not necessarily occur in the presence of oocyte-derived mtDNA (figure 5). This finding may represent a step forward in overcoming the histocompatibility problem of stem cell therapy [47].

These studies demonstrated that cells derived from nuclear transfer can be successfully harvested, expanded in culture, and transplanted *in vivo *with the use of biodegradable scaffolds on which the single suspended cells can organize into tissue structures that are genetically identical to that of the host. These studies were the first demonstration of the use of therapeutic cloning for regeneration of tissues *in vivo*. Others in the field have created mouse SCNT derived c-kit-positive stem cells to restore infarcted myocardium [61], dopaminergic neurons to correct the phenotype of a mouse model of Parkinson disease [62]. The first HESC line derived from SCNT was created in February, 2004 [63].

### Parthenogenesis

Parthenogenesis (<*Gr*. "virgin birth") is production of offspring by a female with no genetic contribution from a male and without meiotic chromosome reduction. The process is common reproductive strategy among insects such as aphids, flies, ants, and honeybees, but is also known to occur in vertebrates including lizards, snakes, fish, birds, and amphibians. The first demonstration of artificially-stimulated parthenogenesis *in vitro *was made by Jacques Loeb (1899), who was able to activate oocytes from sea urchins and frogs by pricking them with a needle or by changing the ambient salt concentration. Pincus (1939) demonstrated parthenogenetic activation of mammalian eggs using temperature and chemical stimuli. Thus far, parthenogenetic activation of eggs has been studied in a variety of mammals including mice, goats, cows, monkeys, and humans. Plachot *et al*. described parthenogenesis in humans by examining 800 human oocytes and showed that 12 activated parthenogenetically and four underwent normal cleavage[64]. Although there have been no reports of naturally-occurring human parthenotes, a human parthenogenetic chimera has been described [65]. The juvenile patient presented with developmental delay, apparent sex reversal, and entirely parthenogenetic blood leukocytes. This finding confirmed the viability of chimeras in higher mammals as presaged by successful murine experiments over the previous two decades (see below).

There is no confirmed example of *de novo *mammalian parthenogenetic reproduction, but mammalian oocytes can be artificially induced to undergo parthenogenesis *in vitro *by a two-step protocol involving electroporation and/or treatment with a chemical agent (ionomycin, ethanol, or inositol 1,4,5-triphosphate) to elevate Ca^2+ ^levels transiently, followed by application of an inhibitor of protein synthesis (cycloheximide) or protein phosphorylation (6-dimethylaminopurine). Success rates and viability appear to be organism dependent. Mouse parthenotes are capable of developing beyond the post-implantation stage *in vivo *[66,67]; porcine parthenotes have developed up to post-activation day 29 (limb bud stage, past the early heart beating stage); rabbit parthenotes until day 10–11 [68]; primates (*Callithrix jacchus*) have only been shown to implant [69]. The reason for this arrested development is believed to be due to genetic imprinting. In normal zygotes maternal and paternal haploid genomes are epigenetically distinct, and both sets are required for successful development [70,71]. Indeed, unstable chromosome modifications in the form of DNA methylation or histone modification are distinctly different in human sperm, compared to eggs. Therefore each gamete carries unique patterns of gene expression into the embryo. Since all genetic material in parthenotes is of maternal origin, there is no paternal imprinting component and this prevents proper development of extraembryonic tissues whose expression is regulated by the male genome [72]. In most mammals – including primates – oocytes are arrested at metaphase II just before ovulation. Cytogenetic microscopy shows the presence of a *2n *polar body under the zona pellucida and a *2n *protonucleus in the cytoplasm. After chemical activation to mimic the effects of sperm penetration on changes in cellular Ca^2+ ^gradient, the cell fails to complete meiosis II. Instead, the second polar body is never extruded, resulting in a diploid protonucleus derived from two sets of sister chromatids. These chromatids then begin to undergo mitosis resulting in a parthenote manifesting uniparental disomy. Although the derivation of embryonic-like stem cells from oocytes (parthenogenetic stem cells, PSC) is relatively inefficient (perhaps due to complexities of genomic imprinting), when they are differentiated into adult tissues, they appear fully functional.

In spite of non-viability of monkey parthenotes, the extracted stem cells seem to assume the morphology and functional behavior of HESC and express appropriate ESC markers. They have embryonic-like replicative ability and have been propagated *in vitro *in an undifferentiated state for up to 14 months. *In vitro*, they have been differentiated into cardiomyocyte-like cells, smooth muscle, beating ciliated epithelia, adipocytes, several types of epithelial cells, as well as dopaminergic and serotoninergic neurons. Almost all of these neurons express TUJ1 (beta-tubulin III), and up to 25% of the TUJ1+ cells co-express tyrosine-hydroxylase. This latter enzyme marker is considered diagnostic for catecholaminergic neurons (dopamine, norepinephrine, and epinephrine [73]). Furthermore, HPLC analysis of culture media following a depolarizing KCl-buffer identifies the release of the neurotransmitters dopamine and serotonin from the cells. Ater two weeks of differentiation, about half of the cells demonstrate neuronal morphology and begin to express voltage-dependent sodium channels that can be blocked by tetrodotoxin.

These observations are recapitulated *in vivo*, since injection of monkey PSC into immunocompromised mice induces formation of benign teratomas containing tissue derivatives from all three germ layers (ectoderm, endoderm and mesoderm) including cartilage, muscle, bone, neurons, skin, hair follicles, and intestinal epithelia [74,75]. Of particular note is the apparent tendency of these cells to differentiate into neuronal tissues, as has been noted by chimera studies [67]. The reasons for this underlying preference are not well understood although one possible explanation is that it is a consequence of purely maternal genomic imprinting, reflecting a lack of epigenetic balance that would be conferred by paternally-imprinted genes.

To be sure, parthenotes are not free from ethical controversy and are viewed by some in society as artificial entities that in some sense represent 'tampering with nature.' Since a parthenote is analogous to a mature ovarian teratoma (a spontaneous *in vivo *tumorigenic event) the *de facto *acceptance of experiments using teratoma tumor tissue lends some legitimacy to experimentation on parthenotes. These contradictions await reconciliation in a comprehensive ethical framework.

### Stem cell genomics

The pluripotentiality of stem cells is also their limitation, and explains why they are not used clinically today. Although ESC can be differentiated into skin, neurons, blood, cardiac cells, cartilage, endothelial cells, muscle, hepatocytes, and pancreatic cells, the efficiency can be quite limited for certain cell types. Another difficulty is studying the quality of differentiation: are the neurons derived from stem cells bona fide neurons, or merely neuronal-like cells? To address this question we developed high throughput methodologies using microarrays to evaluate new stem cell derivatives [76]. We differentiated HESC into retinal pigmented epithelial cells (RPE) (the site of lesions in macular degeneration and retinitis pigmentosa) and used microarrays to identify their genetic signature. We then compared their gene signature to those derived from two established RPE cell lines (one of which has been successfully used clinically). A bronchial epithelial cell line served as a negative control and a freshly isolated human RPE served as a positive control. We demonstrated similarity between our HESC derived RPE and the freshly isolated RPE. The bronchial epithelial and two other established RPE lines were less similar. Interestingly, the data set that represented the genes common to freshly isolated RPE and HESC derived RPE (but not in the two established lines), contained many retinal specific genes. This finding provided further support of the benefits of HESC: the ability to generate a limitless number of HESC with the potential to differentiate along specific lineages to allow creation of RPE cells in quantities necessary for clinical transplantation. The next step would be to couple this technology to ESC derived from SCNT (or parthenogenesis) to create the ideal treatment for macular degeneration and retinitis pigmentosa.

Another technology currently under development at our institution is "genomics guided tissue engineering." Here we perform microarrays periodically during stem cell differentiation. For example, microarrays are performed on undifferentiated monkey PSC, PSC derived neural precursors (PSC-NP), and NP that were further differentiated for 8 days (PSC-neurons). We have identified numerous targets such as receptors and ligands present at each of these distinct time points, and are modifying our culture system in order to improve the quality and quantity of differentiation. Furthermore, we are comparing the gene expression profiles of PSC derived neurons to gene expression profiles of reference neurons. Not only will this provide new insight into the type of neurons that may be generated, but it offers clues into what our stem cell derived neurons might be lacking. We can then go back to the culture system and try to target these specific genes/signaling pathways.

Further study of stem cell genomics will give additional insight into pluripotentiality. An understanding of pluripotentiality might allow for a somatic cell to be de-differentiated into an intermediate stage, which could then be expanded, differentiated and transplanted back into the patient. We are presently characterizing the genetic signature of pluripoteniality by analyzing gene expression among primate stem cells derived from a variety of methods (IVF, parthenogenesis, and adult stem cells). By identifying "stemness" genes by comparing undifferentiated stem cells to their differentiated counterpart, and comparing this to stem cells of different origins, a core set of pluripotential target genes may be mapped. Of particular interest are the 1,075 genes that are similarly down-regulated in IVF derived human ESC and monkey PSC. Furthermore, we have detected paternally imprinted genes in our HESC but not in our PSC data sets. From this we conclude that paternal imprinting might not be necessary for pluriopotentiality.

## Conclusion

Our systems biology approach incorporates the fields of genomics, cell biology, nuclear transfer, and materials science, and utilizes personnel who have mastered the techniques of bioinformatics, cell harvest, culture, expansion, transplantation, as well as polymer design essential for the successful application of these technologies. Experimental efforts are currently underway involving virtually every type of tissue and organ of the human body. Various tissues are at different stages of development with some already being used clinically, a few in pre-clinical trials, and some in the discovery stage. Recent progress suggests that engineered tissues may have an expanded clinical applicability in the future and may represent a viable therapeutic option for those who require tissue replacement or repair.
